# Talker identification under adverse auditory conditions-The impacts of noise, channel, language, and familiarity

**DOI:** 10.1371/journal.pone.0339396

**Published:** 2026-02-23

**Authors:** Ningxue Fan, Puyang Geng, Zhijun Li, Hong Guo

**Affiliations:** 1 Information Security and Social Management Innovation Lab, Shanghai Open University, Shanghai, China; 2 Department of Audio, Video, and Electronic Forensics, Academy of Forensic Science, Shanghai, China; 3 Shanghai Forensic Service Platform, Key Laboratory of Forensic Science, Ministry of Justice; LSU Health Shreveport, UNITED STATES OF AMERICA

## Abstract

**Purpose:**

Talker identification is a crucial auditory skill that underpins human social communication and forensic applications. However, real-world conditions pose several challenges-such as environmental noise, channel variability, language familiarity, and talker familiarity-that can undermine the accuracy of auditory identification. In light of the limitations and insights from previous studies, the present study employed auditory experiments to systematically examine the impact of these four adverse factors on talker identification.

**Methods:**

The study aimed to address two questions: (1) whether the independent and interactive effects among these factors are significant, and (2) whether lab-training can enhance talker identification accuracy. Using a voice line-up paradigm, this study conducted a perception experiment where speech stimuli were presented under four primary conditions: noise (No Noise vs. Noise), channel (High-quality vs. High-quality; Landline vs. Landline, High-quality vs. Landline), language (Mandarin, Reversed Mandarin, English, Reversed English), and speaker familiarity (assessed through listening tests and lab training). Auditory responses to the stimuli under these adverse conditions were collected from 53 listeners.

**Results:**

The findings indicate that environmental noise and channel variability have significantly negative effects on talker identification, while intelligible speech yields superior performance under adverse conditions compared to unintelligible reversed speech. Furthermore, the study found that lab-training (i.e., increasing talker familiarity) could enhance talker identification accuracy under adverse conditions, although it does not improve accuracy under no noise and high-quality channel conditions.

**Conclusion:**

This paper systematically examines the interactive effects of multiple adverse factors on talker identification, thereby advancing our understanding of the auditory mechanisms underlying human social speech communication and providing important theoretical support for auditory examination techniques in forensic speaker identification.

## 1. Introduction

Talker identification is a crucial auditory skill underpinning human social communication, from the early recognition of caregivers in infancy to the complex interactions of adulthood [1,2], and extending to its significant role in forensic applications [3]. In forensic contexts, reliable voice identification is essential for judicial proceedings, drawing on both the naturalistic judgments of ear witnesses and the systematic evaluations conducted by forensic experts [3,4]. However, real-world conditions introduce several challenges-such as environmental noise, channel variability, language familiarity, and talker familiarity-that can undermine the accuracy of auditory identification. Consequently, examining the impact of these adverse auditory conditions is critical not only for refining cognitive models of speech processing but also for advancing effective and robust applications in both communicative and forensic settings.

### 1.1 Effect of noise on talker identification

Research has confirmed that environmental noise significantly disrupts critical acoustic cues [5,6], thereby potentially impairing talker identification accuracy. However, although the number of studies is limited, existing research yields inconsistent results regarding the impact of noise on talker identification. It has been reported that, in three distinct noise environments (i.e., speech-shaped noise, multi-talker babble, and a single, unfamiliar competing talker), the identification accuracy declined as the signal-to-noise ratio (SNR) decreased across all noise conditions, with the most pronounced reduction occurring under multi-talker babble conditions [7]. Similarly, Mamun et al. [8] found that both cochlear implant users and healthy control groups experienced significant declines in talker identification accuracy when exposed to speech-shaped noise.

Other studies highlighted a more complex influence of noise, while aged and hearing-impaired female listeners did not show significant changes under noise or competing talker conditions, hearing-impaired male listeners were significantly affected [9,10]. Furthermore, Kanber et al. [11] found that in a four-talker babble environment, there was no significant difference in identification accuracy between familiar and unfamiliar talkers, with both conditions averaging around 80% accuracy. A review of previous studies also indicates that, regardless of whether the listener is normally hearing or hearing-impaired, and regardless of the familiarity of the talker, identification accuracy rarely exceeds 90% under various noisy conditions [8–11].

### 1.2 Effect of channel variability on talker identification

Channel variability is another common factor influencing daily speech communication and forensic talker identification (e.g., recordings from landline phones and high-definition mobile phones). One fundamental impact of landline phone use, for instance, is its limited frequency range of 400–3400 Hz [12]; this restriction can affect the transmission of crucial acoustic cues, such as F0 and formant frequencies below 400 Hz and above 3400 Hz [3,13], thereby potentially compromising accurate talker identification. However, only a few studies have examined this factor to date. It is found that the channel (i.e., landline vs. mobile phones) significantly affected talker identification accuracy (i.e., approximately 74%) with its negative impact surpassing that of language and dialect (i.e., 81%−86%) [3].

Moreover, the authors pointed out that research on multi-factor interactions in talker identification (e.g., various languages mixed with channel variability) remains extremely scarce. One of the latest studies further revealed that consonant-based talker identification is not affected by channel variability (i.e., full-band, telephone-band, and non-telephone-band recordings), whereas vowel-based talker identification is significantly influenced by the channel [14].

### 1.3 Effect of language familiarity on talker identification

The language familiarity effect is one of the most popular and controversial topics in talker identification research, and it is a key focus of the current study. The central debate in the extant literature concerns whether language intelligibility exerts an influence on talker identification. Specifically, researchers have questioned whether talker identification necessitates language comprehension [15] or whether it can be accomplished without an understanding of the language [16].

The argument in favor of language-independent talker identification originally emerged from early neuropathological research. For instance, patients with receptive aphasia-characterized by impaired language comprehension-can still recognize speakers, whereas patients with phonagnosia lose the ability to identify talkers despite intact ability of language comprehension [17]. Fleming et al. [16] further substantiated this perspective through a perceptual experiment employing backward Chinese and English sentences. Their findings indicated that, although the reversed sentences were largely unintelligible, native English speakers did not exhibit significant cross-language differences in talker identification accuracy; in other words, enhanced familiarity with English phonology did not translate into improved identification performance. Other study has reported similar finding that no significant difference was observed in a talker similarity rating task based on forward and backward speech [18].

Conversely, using a paradigm similar to that of Fleming et al. [16], Perrachione et al. [19] reported results that strongly suggest talker identification is contingent upon language comprehension. In support of this view, several studies involving infants, individuals with dyslexia, and second-language learners have demonstrated that auditory talker identification is facilitated by language comprehension; that is, listeners are generally more adept at discriminating between speakers when the linguistic context is familiar [15,20–22]. Mary Zarate et al. [23] extended this line of inquiry by examining talker identification among native English speakers using a range of stimuli, including non-linguistic sounds, Chinese, German, pseudo-English, and English. Their results revealed a progressive improvement in identification accuracy correlating with increased language familiarity (non-linguistic < Chinese < German < pseudo-English < English). Similarly, other studies demonstrated that talker identification accuracy was significantly higher for rhyming word pairs (e.g., “day-bay”) compared to unrelated word combinations (e.g., “day-bee”), thereby underscoring the role of phonological familiarity [24,25].

### 1.4 Effect of speaker-familiarity/training on talker identification

Another factor influencing talker identification is speaker familiarity. In recent years, researchers have examined the impact of familiarity by comparing the performance of listeners with familiar versus unfamiliar speakers and by employing lab-training paradigms. The majority of studies report that listeners demonstrate significantly higher accuracy when identifying familiar voices compared to unfamiliar ones [26–30]. Nevertheless, even though familiar talkers are identified more accurately, listener performance is not invariably flawless [27,28]. One plausible explanation for the speaker familiarity effect is that listeners are able to extract distinctive acoustic features or leverage prior knowledge associated with familiar speakers [31].

Furthermore, the potential of lab-training to enhance talker identification accuracy has only recently attracted attention over the past two decades. Several investigations have demonstrated that perceptual training can lead to improvements in talker identification accuracy [32–34]. Kanber et al. [11] compared the recognition accuracy among personally familiar voices, lab-trained voices, and unfamiliar voices, and found that brief training (i.e., 5–10 minutes) was sufficient to enhance identification performance. In contrast, other study reported that training does not consistently yield improvements in talker identification; specifically, training benefits observed with foreign-language talkers were restricted to the trained speaker set and did not generalize to novel foreign-language voices [35]. Similarly, McLaughlin et al. [36] found no significant enhancement in talker identification accuracy following training in conditions involving an unfamiliar language.

### 1.5 The present study

In summary, existing research on talker identification under adverse conditions (i.e., environmental noise, channel variability, language familiarity, and talker familiarity) remains limited in quantity, and the findings continue to be contentious. While the effects of these four adverse factors have been investigated individually, to the best of our knowledge based on the current literature, their combined influence on talker identification has yet to be examined. Consequently, in light of the insights and gaps in the current literature, the present study aims to address two primary questions:

(1) What are the individual and interactive effects of noise, channel variability, and language familiarity on talker identification?
(2) Can lab-training designed to enhance talker familiarity improve talker identification accuracy under adverse conditions?

## 2. Method

The research was approved by the Committee for the Protection of Human Subjects (CPHS) at the Academy of Forensic Science (Shanghai, China) [No. 2023−15]. All participants were informed about the study’s purpose, provided written consent form, and received financial compensation after completion of the experiment. Participants were informed that they could withdraw from the experiment at any time if they chose to discontinue their participation. All participants involved in the current study were recruited to participate in this experiment between March and April 2025.

### 2.1 Participant

A preliminary power analysis was conducted via the *pwr* package in R [37,38]. It indicated a sample size > 21.10 was needed to detect a large effect size (Cohen’s *f* = 0.4; [39]), with a significance level of 0.05 and statistical power of 0.80. Consequently, a total of 53 native Mandarin speakers (33 females, 20 males) participated in this study. All participants were undergraduate or graduate students recruited from some universities in China. All participants used English as their second language and had passed the CET-4 (College English Test), indicating an intermediate level of English proficiency. Additionally, none of the participants had received professional auditory training (e.g., musical training) that might bias their auditory perception. The female participants had a mean age of 26.21 years (SD = 3.36), and the male participants had a mean age of 27.36 years (SD = 6.74). None of the participants reported a history of speech or hearing impairments. Upon completion of the study, participants were provided with appropriate financial compensation.

### 2.2 Stimuli

Four female native speakers aged from 31 years to 38 years (SD = 3.16) were recruited to record the speech stimuli for this study. All speakers are fluent in standard Mandarin. They use English as their second language, and each has passed the CET-4, indicating intermediate English proficiency. Additionally, none of the speakers have a history of speech or hearing impairments.

As shown in Table 1, eight target sentences were constructed in both Chinese and English versions, with each sentence comprising 4–11 words. All speech stimuli were recorded in a sound-attenuated room using a high-quality digital recorder (i.e., SONY PCM-D100). Additionally, during a telephone call initiated from an iPhone 14 Pro Max, simultaneous recordings were acquired using a landline telephone (i.e., Motorola C7501RC). The digital recorder and the iPhone 14 Pro Max were positioned 30 cm from the speakers’ mouths. Prior to recording, the speakers were given ample opportunity to familiarize themselves with the materials and practice as needed. They were instructed to articulate each target sentence in their habitual neutral voice twice. Considering the natural variability in a speaker’s acoustic features even when uttering identical content [40], and to maintain ecological validity with daily communication and forensic contexts, different rounds of utterances were used if two sequentially presented speech stimuli originated from the same speaker. All recordings were saved in WAV format at a 44.1 kHz sampling rate and 16-bit resolution. In total, 4 (speakers) * 8 (target sentences) * 2 (Chinese and English) * 2 (times) * 2 (digital recorder and landline phone) = 256 recordings were collected.

**Table 1:** Target sentences in Chinese and English versions.

No.	Target sentences in Chinese	Target sentences in English
1	/mɪŋ2//tʰjɛn1//i4//tɕʰi3//tɕi1//fan4/	Let’s have a meal together tomorrow.
2	/pa1//tjɛn3//kʰɤ3//i3//ʈʂʰu1//fa1/	We can depart at eight o’clock.
3	/uɔ3//ɕja4//u3//tɕʰy4//na2//kʰuaɪ4//ti4/	I will go to pick up the package in the afternoon.
4	/tʰa1//ʈʂɤ4//tsʰɯ4//ʈʂʰu1//ʈʂʰa4//meɪ2//taɪ4//uɔ3/	He didn’t take me on this business trip.
5	/uɔ3//meɪ2//uən4//tʰi2/	I have no problem.
6	/tsaɪ4//pu2//tɕʰy4//tɕjoʊ4//wan3//lɤ0/	If we don’t go soon, we will be late.
7	/tʰa1//tsweɪ4//tɕɪn4//tɕɪŋ1//ʈʂʰɑŋ2//pu2//tsaɪ4/	He has been frequently absent recently.
8	/ʈʂɤ4//ɕja4//uɔ3//kʰɤ3//i3//xwan4//ʂoʊ3//tɕi1//lɤ0/	Now I can get a new phone.

The speech stimuli were firstly normalized to 70 dB and subsequently reversed using Praat software [41]. Consequently, four categories of speech stimuli (i.e., Mandarin, Mandarin-reverse, English, English-reverse) were created to examine the influence of language familiarity on talker identification. These stimuli were then divided into two groups to assess the impact of channel variability. Specifically, those recorded using the digital recorder (including both forward and reversed versions) were designated as High-quality (H), while the recordings obtained via the landline telephone were labeled as Landline (L).

To further investigate the effects of noise on talker identification, high-quality speech stimuli across all four categories were synthesized with a mixed noise component, following a methodology analogous to that employed in previous speech-in-noise perception tasks (e.g., [42,43]). Previous studies have frequently employed sine waves and broadband noise (e.g., white noise) in the investigation of speech-in-noise perception, revealing that both exert a masking effect on the transmission of speech information [44–48]. To emulate as closely as possible the impact of noise on speech perception in realistic interference scenarios, the present study generated a composite noise signal by combining sine waves and white noise, employing the default formula integrated within Praat (i.e., 1/2 * sin (2π × 377 × x) + randomGauss (0, 0.1)) at a sampling rate of 44.1 kHz. For all speech materials under noise conditions, the signal-to-noise ratio (SNR) was maintained at 0 dB.

To examine the influence of speaker familiarity on talker identification, a lab-training paradigm was employed in the auditory perceptual experiment. All speech stimuli from the speakers were divided into two sessions (1–4 target sentences for the listening test; 5–8 target sentences for the lab-training test). For the listening test, stimuli representing the four categories (i.e., Mandarin, Mandarin-reverse, English, English-reverse) under adverse noise (i.e., No Noise vs. Noise) and channel conditions (i.e., High-quality vs. High-quality; Landline vs. Landline; High-quality vs. Landline) were utilized. In the lab-training test, listeners were firstly exposed to the four categories of speech stimuli in adverse noise and channel conditions from a single speaker twice, after which they completed a perceptual talker identification task for that speaker. This procedure was conducted sequentially for all four speakers. The speech stimuli of the two sessions (i.e., listening test and lab-training test) for talker identification experiment were shown in Table 2.

**Table 2:** The speech stimuli of the two sessions (i.e., listening test and lab-training test) for talker identification experiment.

Session	Target sentence	Language	Channel	Noise	Identity
Listening test	1-4	Mandarin Mandarin-reverse English English-reverse	High-quality vs. High-quality	No Noise vs. Noise	Same/Different
		Mandarin Mandarin-reverse English English-reverse	Landline vs. Landline High-quality vs. Landline	/	Same/Different
Lab-training test	5-8	Mandarin Mandarin-reverse English English-reverse	High-quality vs. High-quality	No Noise vs. Noise	Same/Different
		Mandarin Mandarin-reverse English English-reverse	Landline vs. Landline High-quality vs. Landline	/	Same/Different

It is important to note that, to limit experimental sessions to approximately 40 minutes and maintain participant engagement and attention, this stimulus set of the current study has several limitations. For instance, it only included four female talkers, used a relatively high signal-to-noise ratio (SNR = 0), and featured just two training rounds. These limitations necessitate caution when interpreting the study’s results, as they may constrain the generalizability of the findings. Nevertheless, the study systematically examines interactions among multiple adverse factors in talker identification, offers key insights into auditory talker identification patterns under complex adverse conditions, and lays groundwork for understanding how listeners process talker information amid combined auditory challenges. Future research can build on these findings by conducting more targeted, comprehensive investigations to address these constraints.

### 2.3 Procedure

The perceptual experiment was conducted in a sound-attenuated room. Each participant was instructed to sit in front of a laptop monitor and adjust the screen to a position that allowed clear visibility. Professional high-quality headphones (Sennheiser HD650 and Audio-Technica ATH-M70x) were used in the perceptual experiment. The experiment was conducted with PsychoPy software [49].

**Fig 1:**
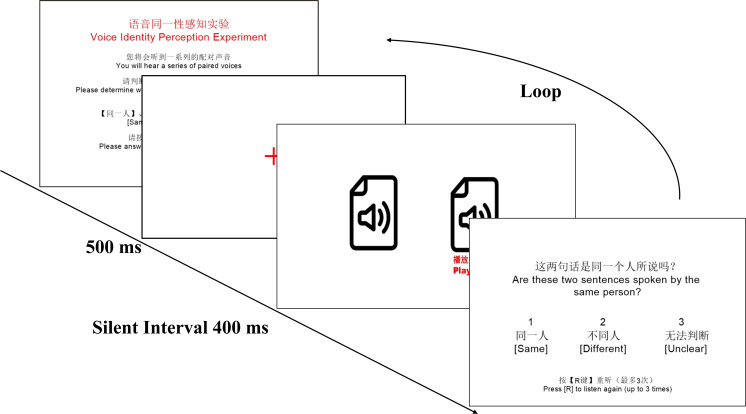
The procedure of perceptual talker identification experiment.

The procedure for the perceptual talker identification experiment was illustrated in Fig 1. For listening test session, each trial began with a 500-millisecond red fixation cross. Subsequently, two stimuli (i.e., either from the same speaker or from different speakers) were presented in a voice line-up paradigm, separated by a 400-millisecond silent interval. Participants were then required to select one of three response options (i.e., Same, Different, or Unclear) based on the stimuli they heard. For the lab-training test session, the procedure commenced with two rounds of auditory training using the speech stimuli from a single speaker (as shown in Table 2). Following the training phase, participants engaged in a talker identification task that followed the same procedure as the listening test session. The entire experiment lasted approximately 30–45 minutes. To mitigate auditory fatigue, participants were permitted to take breaks at any time during the session. Before the formal data collection, participants were provided with instructions for the perceptual experiment. They also completed two practice trials to familiarize themselves with the experimental procedure. Subsequently, perceptual data for each stimulus were collected from all 53 participants.

### 2.4 Data analysis

Two generalized logistic regression analyses were conducted for the listening test session using the *afex* package [50] in R software [38] to investigate the impact of adverse auditory conditions on talker identification accuracy. In these models, each stimulus’s perceptual judgment was re-coded as a binary outcome (0 for an incorrect response, 1 for a correct response; “unclear” responses were excluded from the analysis) and served as the dependent variable. For one model, the independent variables were noise (No Noise vs. Noise) and language (Mandarin, Mandarin-reverse, English, English-reverse); for the other, they were channel (High-quality vs. High-quality; Landline vs. Landline; High-quality vs. Landline) and language. The models were constructed using the following formulas: Answer ~ Noise * Language + (1 | Speaker) + (1 | Listener); Answer ~ Channel * Language + (1 | Speaker) + (1 | Listener).

Furthermore, two additional generalized logistic regression analyses were performed to assess the effect of lab-training on talker identification under adverse auditory conditions. In these analyses, perceptual accuracy, coded as 0 or 1, was the dependent variable. For one model, the independent variables were familiarity (Listening test vs. Lab-training test), noise, and language; for the other, they were familiarity, channel, and language. These models were specified as follows: Answer ~ Train * Noise * Language + (1 | Speaker) + (1 | Listener); Answer ~ Train * Channel * Language + (1 | Speaker) + (1 | Listener).

In all models, the random intercepts for speakers and listener as well as the random slope for noise, channel, and language by the listener were included in all models to support the maximal random effect structure design [51]. The likelihood ratio test was used to assess the importance of the random slope, which indicated that the slope was not significant in any of the model fittings. Consequently, to maintain model simplicity, the random slope was removed from all models. Tukey’s HSD post hoc tests were subsequently performed for pairwise comparisons [52], and odds ratios were reported as the measure of effect size.

## 3. Results

**Fig 2:**
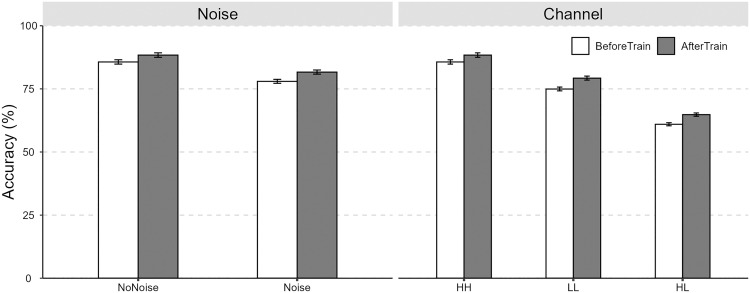
The perceptual accuracies (±95% CI) of talker identification under noise conditions and channel conditions across the two sessions.

**Fig 3:**
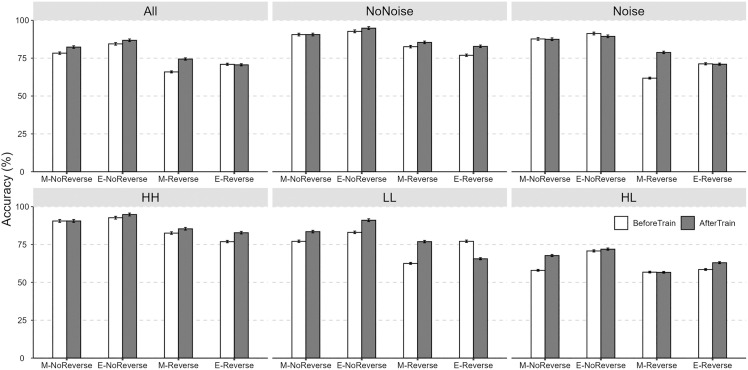
The perceptual accuracies (±95% CI) for talker identification under language conditions as well as the accuracy under the noise × language and channel × language interactions across the two sessions.

The average accuracies for the talker identification task under noise (No Noise vs. Noise), channel (High-quality vs. High-quality [HH]; Landline vs. Landline [LL]; High-quality vs. Landline [HL]), and language conditions (Mandarin [M], Mandarin-reverse [M-reverse], English [E], English-reverse [E-reverse]) across the two sessions (Listening Test vs. Lab-training) are presented in Figs 2 and 3. As shown in Table 3, a raw comparison of the statistical results indicated that noise, poorer signal transmission (i.e., Landline vs. Landline), and channel discrepancies (High-quality vs. Landline) all resulted in reduced talker identification accuracy. Although forward speech yielded significantly superior talker identification performance compared to backward speech, no language familiarity effect was observed (i.e., talker identification accuracies were comparable for Mandarin and English). Additionally, lab-training (i.e., higher speaker familiarity) moderately improved talker identification accuracy. In terms of response times, longer identification times were observed under conditions of poorer signal transmission and channel difference conditions. Following lab-training, response times decreased across all conditions.

**Table 3:** The average accuracies and reaction times of talker identification task under adverse auditory conditions.

Speaker familiarity	Factors		Accuracy (%)	Reaction Time (milli-second)
Listening test	Channel	HH	85.66	907.54
		LL	74.94	973.48
		HL	60.98	1066.05
	Language	M	78.31	1040.63
		M-Reverse	65.90	971.65
		E	84.42	851.70
		E-Reverse	70.93	945.25
	Noise	No Noise	85.66	907.54
		Noise	77.97	861.95
Lab-training test	Channel	HH	88.38	698.81
		LL	79.25	732.57
		HL	64.80	862.87
	Language	M	82.30	708.04
		M-Reverse	74.41	768.31
		E	86.79	729.54
		E-Reverse	70.58	830.30
	Noise	No Noise	88.38	698.81
		Noise	81.65	741.96

To further illustrate the impact of these adverse auditory conditions on talker identification, two generalized logistic regression models were conducted. These results of the models (as shown in S1 Appendix) revealed significant main effects of “Noise”, “Channel” and “Language”, as well as significant two-way interaction effects of “Noise × Language” and “Channel × Language” (*p* < 0.05) on talker identification accuracy. In instances where a higher-order interaction effect was significant, the corresponding main effects and lower-order interaction effects were not interpreted.

As shown in S1 Appendix, the results of Tukey-HSD post hoc analysis for the two-way interaction effect of “Noise × Language” demonstrated that, (1) for reversed speech, talker identification accuracies were significantly higher in the no noise condition than in the noise condition, while for forward speech, there was no significant difference in talker identification accuracies between the no noise and noise conditions; (2) forward speech yielded higher identification accuracies than reversed speech under both the no noise and noise conditions. Additionally, significantly lower talker identification accuracy was observed for reversed Mandarin speech under the noise condition compared to reversed English.

The results of the Tukey HSD post hoc analysis examining the two-way interaction effect of “Channel × Language” are presented in S1 Appendix. Overall, talker identification accuracy was highest for the High-quality vs. High-quality condition. In addition, identical channel conditions (i.e., High-quality vs. High-quality and Landline vs. Landline) generally yielded higher accuracy than mismatched channel conditions (i.e., High-quality vs. Landline) across all language conditions, with two exceptions: no significant difference was observed for reversed English between the High-quality vs. High-quality and Landline vs. Landline conditions, and for reversed Mandarin between the High-quality vs. Landline and Landline vs. Landline conditions. Furthermore, the post hoc analyses revealed that, (1) for High-quality vs. High-quality condition, forward speech exhibited significantly higher identification accuracy than reversed speech; (2) for Landline vs. Landline condition, reversed Mandarin speech showed lower accuracy than the other three language conditions; and (3) for High-quality vs. Landline condition, English speech showed higher accuracy than the other three language conditions.

Considering the impact of speaker familiarity on talker identification, the results from two generalized logistic regression models are shown in S1 Appendix. These models identified significant main effects of “Familiarity”, “Noise”, “Channel” and “Language”, significant two-way interaction effects of “Familiarity × Language” and “Channel × Language”, and significant three-way interaction effects of “Familiarity × Noise × Language” and “Familiarity × Channel × Language” (*p* < 0.05) on talker identification accuracy.

The Tukey HSD post hoc analysis for the three-way interaction of “Familiarity × Noise × Language” revealed that talker identification accuracy was significantly higher in the Lab-training session compared to the Listening test session only for reversed English in the no noise condition {*β* = 0.40, *SE* = 0.18, *t* = 2.22, *p* = 0.03, OR = 1.45 (95% CI: [1.03, 2.03])} and reversed Mandarin in the noise condition {*β* = 0.90, *SE* = 0.16, *t* = 5.60, *p* < 0.001, OR = 2.30 (95% CI: [1.70, 3.12])}.

Furthermore, as shown in S1 Appendix, the Tukey HSD analysis for the three-way interaction of “Familiarity × Channel × Language” indicated that the improvement in identification accuracy following lab-training (i.e., higher speaker familiarity) was observed exclusively in the poor signal transmission condition (i.e., Landline vs. Landline), with the exception of English speech. Additionally, a positive impact of speaker familiarity was found for Mandarin speech in the High-quality vs. Landline condition and for reversed English speech in the High-quality vs. High-quality condition.

## 4. Discussion

The current study aims to investigate talker identification under adverse auditory conditions and whether lab-training can enhance listeners’ performance. Through perceptual experiments of talker identification conducted in two sessions (i.e., Listening Test and Lab-training Test), the study found that adverse auditory conditions, specifically environmental noise, channel variability, and speaker familiarity, have a significant impact on talker identification. Moreover, although language familiarity did not have a significant effect on talker identification, forward speech yielded significantly higher identification accuracy compared to reversed (i.e., unintelligible) speech.

### 4.1 Complex interactive effects of adverse conditions on talker identification

In experiments involving speech mixed with noise, this study found that noise exerted a significant adverse effect only on reversed speech, with no such effect observed for forward speech. This finding contrasts with studies on forward speech, which have reported a decline in identification accuracy due to noise [7,8], but it further supports the view that noise exerts a complex influence on talker identification [10,11]. In light of evidence suggesting that reversed speech does not facilitate the formation of short-term memory representations of the speaker for the listener [53–55], we propose a potential hypothesis that talker identification under the reversed speech condition may be more susceptible to noise. Conversely, as listeners are better able to establish short-term memory of the speaker through forward speech, they may experience less interference from noise during talker identification. This hypothesis though requires further systematic auditory and neuroimaging investigations, potentially employing 1-back or n-back experimental paradigms to assess the difficulty of recalling short-term memory for talker identification under conditions of varying speech intelligibility.

The results regarding channel variability support previous fragmented findings [3], showing that talker identification accuracy declines significantly under poor signal transmission (i.e., Landline vs. Landline) and across different channels (i.e., High-quality vs. Landline), following a descending order of High-quality vs. High-quality > Landline vs. Landline > High-quality vs. Landline. Moreover, consistent with previous suggestions that language and channel may exhibit complex interactive effects [3,14], the current study found that in the High-quality vs. High-quality condition, the accuracy for forward speech was superior to that of reversed speech, whereas in the Landline vs. Landline and High-quality vs. Landline conditions, reversed Mandarin and English speech stimuli displayed higher accuracies, respectively. Both the current study and Wang et al.’s work [14] confirm that adverse conditions interact in complex ways rather than through a simple linear summation. This finding underscores the need for future research to build upon these fragmented observations and to conduct more systematic, in-depth investigations into the interactive effects between language and channel.

Surprisingly, this study did not find a significant language familiarity effect on talker identification. Despite being one of the most controversial topics in the literature, most studies have reported significant effects of language familiarity [19,21,33]. The current research revealed that, regardless of the presence or absence of noise, forward speech yielded higher talker identification rates than reversed speech; additionally, forward speech in the High-quality vs. High-quality condition outperformed reversed speech, and the speech stimuli from the four language categories (i.e., Mandarin, English, reversed Mandarin, and reversed English) exhibited a complex pattern when in poor signal transmission and different channel conditions. Based on the results of this study, it appears that the intelligibility of language may play a more critical role in talker identification than phonological familiarity [36]. However, the effect of language familiarity on talker identification under varying channel conditions remains complex and warrants further investigation.

### 4.2 Modest improvements of lab-training on talker identification

Consistent with previous studies [32–34], the current study found increased speaker familiarity (after lab-training) could led to modest improvements on talker identification accuracy under adverse auditory conditions (e.g., reversed speech, noise, poor signal transmission, and different channels), with overall gains of approximately 3–4%. Kanber et al. [11] argued that 5–10 minutes of training was sufficient to enhance lab-trained voice identification performance (over 80%). By contrast, the lab-training in the current study yielded only limited improvements in talker identification (see Fig 2), potentially due to the fact that the training comprised only two rounds. Future research could conduct more systematic investigations into how different training durations influence talker identification.

Notably, the current study also found interactive effects between speaker familiarity and noise, channel, and language, as evidenced by the inconsistency of the training effect across conditions. While improvements occurred under adverse conditions, listeners’ accuracy for intelligible speech (no noise, high-quality channels) did not improve with training (see Table 3). This inconsistency confirms training benefits were small and selective, limited to adverse auditory scenarios rather than generalizable.

### 4.3 Implications for forensic speaker identification

In forensic practice, speech is often recorded under varying conditions of noise, channel, and language [56–58]. Auditory examination constitutes a critical component of the acoustic-phonetic paradigm used in forensic speaker identification [4,59]. Therefore, the findings of the current study offer tentative implications for such examinations. First, talker identification is significantly impaired under adverse auditory conditions, which necessitates careful attention to judicial examination procedures. Potential interventions may include speech denoising and signal simulation techniques designed to present speech for identity judgment in conditions that are as optimal as possible [58,60]. Furthermore, when forensic experts encounter unintelligible speech or unfavorable signal conditions, repeated perceptual training to enhance familiarity with the target speaker could yield small improvements in identification accuracy. However, it is important to note that these benefits are not universally observed.

Several limitations of this study warrant discussion. First, this research examined the identification of speech from only four female talkers. Previous studies have reported potential gender differences in talker identification (e.g., male listeners showing higher identification accuracy for male talkers; [61]). Future studies will include additional research on male talkers to confirm these effects. Second, given the repeated mention of the significant application of talker identification in forensic contexts, it remains an interesting topic to explore whether forensic experts differ from untrained listeners. Lastly, to deepen our understanding of the mechanisms underlying talker identification, further research employing neuroscience and brain-imaging techniques is necessary to corroborate the present findings.

## 5. Conclusion

This study discusses the effects of adverse auditory conditions (i.e., environmental noise, channel variability, language familiarity, and speaker familiarity) on talker identification. The findings indicate that both environmental noise and channel variability negatively impact talker identification. In particular, when the channel transmits poor signals or varies in nature, the accuracy of talker identification is significantly reduced. Furthermore, intelligible language demonstrates superior recognition performance under adverse conditions compared to unintelligible language, and this effect appears to be independent of phonological familiarity. Finally, lab-training designed to enhance speaker familiarity moderately improves talker identification accuracy under adverse auditory conditions, while it has no effect on accuracy under no-noise and high-quality conditions. This study systematically examines the interactive effects of multiple factors on talker identification, thereby enriching our understanding of the underlying auditory mechanisms under various auditory conditions and providing important theoretical support for auditory examination techniques in forensic speaker identification.

## Supporting information

S1 Appendix The results of the statistical analysis. (DOCX)

S1 File The datasets analyzed of the current study. (CSV)
