# Supplementary material for: Talker identification under adverse auditory conditions-The impacts of noise, channel, language, and familiarity
Source: PLoS One. 2026 Feb 23;21(2):e0339396. doi: 10.1371/journal.pone.0339396 (PMC12928432; doi:10.1371/journal.pone.0339396)
Supplement: S1 Appendix — (DOCX) [file pone.0339396.s001.docx]

**S1 Appendix.**

**1.** **The results of the two general logistic regression models on the impact of adverse auditory conditions on talker identification.**

| Model | Factor | ***df*** | ***χ^2^*** | ***p*** |
| --- | --- | --- | --- | --- |
| Impact of noise and language | Noise | 1 | 22.55 | **<0.001** |
|  | Language | 3 | 176.14 | **<0.001** |
|  | Noise × Language | 3 | 17.93 | **<0.001** |
| Impact of channel and language | Channel | 2 | 293.36 | **<0.001** |
|  | Language | 3 | 93.26 | **<0.001** |
|  | Channel × Language | 6 | 41.83 | **<0.001** |

**2. The results of Tukey-HSD post hoc analysis on the two-way interaction effect of “Noise × Language” in the general logistic regression model.**

| Noise | Language | *β* | *SE* | *t* | *p* | OR | 95% CI |
| --- | --- | --- | --- | --- | --- | --- | --- |
| No noise-Noise | Mandarin | 0.31 | 0.23 | 1.36 | 0.18 | 1.35 | [0.87, 2.09] |
|  | Mandarin reverse | 1.14 | 0.17 | 6.85 | <0.001 | 2.92 | [2.14, 4.04] |
|  | English | 0.20 | 0.26 | 0.79 | 0.43 | 1.22 | [0.74, 2.01] |
|  | English reverse | 0.31 | 0.16 | 1.95 | 0.05 | 1.34 | [0.99, 1.83] |
| No noise | Mandarin-English | -0.29 | 0.25 | -1.16 | 0.65 | 0.76 | [0.46, 1.23] |
|  | Mandarin-Mandarin reverse | 0.73 | 0.21 | 3.45 | 0.003 | 2.02 | [1.35, 3.08] |
|  | Mandarin-English reverse | 1.10 | 0.21 | 5.38 | <0.001 | 2.88 | [1.95, 4.32] |
|  | English-English revere | 1.39 | 0.22 | 6.29 | <0.001 | 3.81 | [2.51, 5.94] |
|  | English-Mandarin reverse | 1.02 | 0.23 | 4.48 | <0.001 | 2.68 | [1.74, 4.23] |
|  | Mandarin reverse-English reverse | 0.37 | 0.18 | 2.11 | 0.15 | 1.42 | [1.02, 2.00] |
| Noise | Mandarin-English | -0.39 | 0.23 | -1.73 | 0.31 | 0.68 | [0.44, 1.06] |
|  | Mandarin-Mandarin reverse | 1.57 | 0.18 | 8.58 | <0.001 | 4.40 | [3.12, 6.29] |
|  | Mandarin-English reverse | 1.11 | 0.19 | 5.97 | <0.001 | 2.87 | [2.02, 4.14] |
|  | English-English revere | 1.51 | 0.21 | 7.33 | <0.001 | 4.21 | [2.85, 6.34] |
|  | English-Mandarin reverse | 1.96 | 0.20 | 9.70 | <0.001 | 6.45 | [4.41, 9.65] |
|  | Mandarin reverse-English reverse | -0.46 | 0.15 | -3.02 | 0.01 | 0.65 | [0.49, 0.87] |

**3. The results of Tukey-HSD post hoc analysis on the two-way interaction effect of “Channel × Language” in the general logistic regression model. In the table, HH stands for High-quality vs. High-quality, LL stands for Landline vs. Landline, and HL stands for High-quality vs. Landline.**

| Language | Channel | *β* | *SE* | *t* | *p* | OR | 95% CI |
| --- | --- | --- | --- | --- | --- | --- | --- |
| HH | Mandarin-English | -0.28 | 0.25 | 1.13 | 0.67 | 0.69 | [0.49, 0.97] |
|  | Mandarin-Mandarin reverse | 0.71 | 0.21 | 3.39 | 0.004 | 2.02 | [1.35, 3.08] |
|  | Mandarin-English reverse | 1.07 | 0.20 | 5.28 | <0.001 | 2.88 | [1.95, 4.32] |
|  | English-English revere | 1.35 | 0.22 | 6.16 | <0.001 | 3.81 | [2.51, 5.94] |
|  | English-Mandarin reverse | 1.00 | 0.23 | 4.39 | <0.001 | 2.68 | [1.74, 4.23] |
|  | Mandarin reverse-English reverse | 0.36 | 0.21 | 2.07 | 0.17 | 0.50 | [0.37, 0.67] |
| LL | Mandarin-English | -0.38 | 0.18 | -2.16 | 0.13 | 0.76 | [0.46, 1.23] |
|  | Mandarin-Mandarin reverse | 0.72 | 0.15 | 4.66 | <0.001 | 2.02 | [1.50, 2.74] |
|  | Mandarin-English reverse | 0.00 | 0.17 | 0.00 | 1.00 | 2.88 | [1.95, 4.32] |
|  | English-English revere | 0.38 | 0.18 | 2.16 | 0.13 | 3.81 | [2.51, 5.94] |
|  | English-Mandarin reverse | 1.10 | 0.17 | 6.65 | <0.001 | 2.93 | [2.14, 4.06] |
|  | Mandarin reverse-English reverse | -0.72 | 0.15 | *-*4.66 | <0.001 | 0.49 | [0.37, 0.67] |
| HL | Mandarin-English | -0.58 | 0.15 | *-*3.93 | <0.001 | 0.57 | [0.43, 0.76] |
|  | Mandarin-Mandarin reverse | 0.05 | 0.14 | 0.35 | 0.99 | 1.05 | [0.80, 1.38] |
|  | Mandarin-English reverse | -0.02 | 0.14 | 0.17 | 0.99 | 0.98 | [0.74, 1.28] |
|  | English-English revere | 0.55 | 0.15 | 3.77 | <0.001 | 1.72 | [1.29, 2.29] |
|  | English-Mandarin reverse | 0.63 | 0.15 | 4.27 | <0.001 | 1.85 | [1.39, 2.46] |
|  | Mandarin reverse-English reverse | -0.07 | 0.14 | 0.35 | 0.99 | 0.93 | [0.71, 1.22] |
| Mandarin | HH-HL | 1.97 | 0.19 | 10.15 | <0.001 | 6.96 | [4.81, 10.27] |
|  | HH-LL | 1.06 | 0.20 | 5.20 | <0.001 | 2.84 | [1.92, 4.26] |
|  | LL-HL | 0.91 | 0.15 | 5.96 | <0.001 | 2.45 | [1.82, 3.31] |
| Mandarin reverse | HH-HL | 1.31 | 0.16 | 8.04 | <0.001 | 3.61 | [2.64, 4.97] |
|  | HH-LL | 1.06 | 0.16 | 6.49 | <0.001 | 2.84 | [2.07, 3.92] |
|  | LL-HL | 0.24 | 0.14 | 1.72 | 0.20 | 1.27 | [0.97, 1.67] |
| English | HH-HL | 1.67 | 0.22 | 7.78 | <0.001 | 5.24 | [3.48, 8.11] |
|  | HH-LL | 0.96 | 0.23 | 4.23 | <0.001 | 2.59 | [1.68, 4.10] |
|  | LL-HL | 0.72 | 0.17 | 4.24 | <0.001 | 2.02 | [1.46, 2.82] |
| English reverse | HH-HL | 0.88 | 0.15 | 5.73 | <0.001 | 2.36 | [1.76, 3.19] |
|  | HH-LL | -0.01 | 0.16 | -0.08 | 0.99 | 0.99 | [0.72, 1.36] |
|  | LL-HL | 0.89 | 0.15 | 5.81 | <0.001 | 2.39 | [1.78, 3.23] |

**4. The results of the two general logistic regression models on the effect of familiarity on talker identification under adverse auditory conditions.**

| Model | Factor | ***df*** | ***χ^2^*** | ***p*** |
| --- | --- | --- | --- | --- |
| Impact of familiarity, noise, and language | Familiarity | 1 | 7.41 | **0.007** |
|  | Noise | 1 | 52.39 | **<0.001** |
|  | Language | 3 | 261.04 | **<0.001** |
|  | Familiarity × Noise | 1 | 0.34 | 0.56 |
|  | Familiarity × Language | 3 | 9.88 | **0.02** |
|  | Noise × Language | 3 | 6.72 | 0.08 |
|  | Familiarity × Noise × Language | 3 | 12.71 | **0.005** |
| Impact of familiarity, channel, and language | Familiarity | 1 | 20.74 | **<0.001** |
|  | Channel | 2 | 596.27 | **<0.001** |
|  | Language | 3 | 218.43 | **<0.001** |
|  | Familiarity × Channel | 2 | 1.73 | 0.42 |
|  | Familiarity × Language | 3 | 8.98 | **0.03** |
|  | Channel × Language | 6 | 34.66 | **<0.001** |
|  | Familiarity × Channel × Language | 6 | 41.32 | **<0.001** |

**5. The results of Tukey-HSD post hoc analysis on the three-way interaction effect of “Familiarity × Channel × Language” in the general logistic regression model. In the table, HH stands for High-quality vs. High-quality, LL stands for Landline vs. Landline, and HL stands for High-quality vs. Landline.**

| Familiarity | Language | Channel | *β* | *SE* | *t* | *p* | OR | 95% CI |
| --- | --- | --- | --- | --- | --- | --- | --- | --- |
| Lab training test-Listening test | Mandarin | HH | 0.003 | 0.24 | 0.01 | 0.99 | 1.00 | [0.63, 1.59] |
|  |  | LL | 0.42 | 0.18 | 2.36 | **0.02** | 1.50 | [1.07, 2.12] |
|  |  | HL | 0.44 | 0.15 | 2.99 | **0.003** | 1.52 | [1.15, 2.02] |
|  | Mandarin-reverse | HH | 0.22 | 0.19 | 1.14 | 0.26 | 1.24 | [0.85, 1.79] |
|  |  | LL | 1.06 | 0.16 | 6.49 | **<0.001** | 2.00 | [1.48, 2.70] |
|  |  | HL | -0.24 | 0.14 | -1.72 | 0.20 | 1.00 | [0.76, 1.31] |
|  | English | HH | 0.37 | 0.29 | 1.28 | 0.20 | 1.44 | [0.82, 2.56] |
|  |  | LL | 0.75 | 0.22 | 3.47 | **<0.001** | 2.08 | [1.38, 3.18] |
|  |  | HL | 0.06 | 0.15 | 0.39 | 0.70 | 1.06 | [0.79, 1.42] |
|  | English-reverse | HH | 0.38 | 0.18 | 2.17 | **0.03** | 1.45 | [1.03, 2.03] |
|  |  | LL | -0.59 | 0.16 | -3.77 | **<0.001** | 0.57 | [0.42, 0.76] |
|  |  | HL | 0.20 | 0.14 | 1.36 | 0.17 | 1.21 | [0.92, 1.59] |
